# Causal associations between thyroid cancer and IgA nephropathy: a Mendelian randomization study

**DOI:** 10.1186/s12864-023-09633-6

**Published:** 2023-09-05

**Authors:** Ziwei Mei, Fuhao Li, Ruizhen Chen, Zilong Xiao, Dongsheng Cai, Lie Jin, Xu Qian, Yucheng Wang, Jun Chen

**Affiliations:** 1 Zhejiang Chinese Medical University, Hangzhou, Zhejiang 310000 China; 2 Lishui Municipal Central hospital, Lishui, Zhejiang 323000 China; 3 Department of Cardiology, Zhongshan Hospital, Shanghai Institute of Cardiovascular Diseases, Shanghai Medical College of Fudan University, Shanghai, China; 4 Department of Cardiology, Sir Run Run Shaw Hospital, School of Medicine, Zhejiang University, Hangzhou, China; 5 Department of Clinical Laboratory, The Cancer Hospital of the University of Chinese Academy of Sciences (Zhejiang Cancer Hospital), Institute of Basic Medicine and Cancer (IBMC), Chinese Academy of Sciences, Hangzhou, 310022 China

**Keywords:** Thyroid cancer, IgA nephropathy, Mendelian randomization, Genetics

## Abstract

**Background:**

The incidence of kidney disease caused by thyroid cancer is rising worldwide. Observational studies cannot recognize whether thyroid cancer is independently associated with kidney disease. We performed the Mendelian randomization (MR) approach to genetically investigate the causality of thyroid cancer on immunoglobulin A nephropathy (IgAN).

**Methods and results:**

We explored the causal effect of thyroid cancer on IgAN by MR analysis. Fifty-two genetic loci and single nucleotide polymorphisms were related to thyroid cancer. The primary approach in this MR analysis was the inverse variance weighted (IVW) method, and MR‒Egger was the secondary method. Weighted mode and penalized weighted median were used to analyze the sensitivity. In this study, the random-effect IVW models showed the causal impact of genetically predicted thyroid cancer across the IgAN risk (OR, 1.191; 95% CI, 1.131–1.253, *P* < 0.001). Similar results were also obtained in the weighted mode method (OR, 1.048; 95% CI, 0.980–1.120, *P* = 0.179) and penalized weighted median (OR, 1.185; 95% CI, 1.110–1.264, *P* < 0.001). However, the MR‒Egger method revealed that thyroid cancer decreased the risk of IgAN, but this difference was not significant (OR, 0.948; 95% CI, 0.855–1.051, *P* = 0.316). The leave-one-out sensitivity analysis did not reveal the driving influence of any individual SNP on the association between thyroid cancer and IgAN.

**Conclusion:**

The IVW model indicated a significant causality of thyroid cancer with IgAN. However, MR‒Egger had a point estimation in the opposite direction. According to the MR principle, the evidence of this study did not support a stable significant causal association between thyroid cancer and IgAN. The results still need to be confirmed by future studies.

**Supplementary Information:**

The online version contains supplementary material available at 10.1186/s12864-023-09633-6.

## Clinical perspective

### What is new?

Previous studies have discovered some associations between kidney disease and thyroid cancer. However, the causal influence of thyroid cancer on IgAN has not been recognized in observational studies. This MR study provides genetic evidence of the causal link between thyroid cancer and IgAN.

### What are the clinical implications?

These findings do not support the causally increased risk of IgAN induced by thyroid cancer. However, according to the underlying mechanism of circulating immune complexes and complement activation, we cannot ignore the potential connection between IgAN and thyroid cancer. Clinicians and patients should pay great attention to kidney protection in thyroid cancer treatment and prognosis. Exploration and validation of the underlying mechanism will provide valuable guidance for kidney protection.

## Introduction

Thyroid cancer is the most common endocrine malignancy. The incidence of thyroid cancer is 15.7 per 100,000 population per year in the US [1, 2]. Worldwide, there are an estimated 567,233 new cases of thyroid cancer and 41,071 deaths per year [3]. Thyroid hormones influence renal development, the glomerular filtration rate, renal transport systems, and water/electrolyte homeostasis [4]. Thyroid dysfunction also causes kidney disease [5, 6]. Thyroid dysfunction is associated with nephrotic syndrome, including IgAN, membranoproliferative glomerulonephritis, and minimal change disease [7–10].

The relationship between thyroid dysfunction and kidney disease has been investigated for many years. However, the impact of thyroid cancer on kidney injury is less explored. Observational studies have demonstrated the increased risk of renal disease from thyroid cancer [11–13]. This risk is probably related to thyroid cancer treatment and genetic factors [6]. Although some cases reported nephrotic syndrome resulting from thyroid cancer [14], the causal association between thyroid cancer and nephrotic syndrome has not been elucidated in observational studies. IgAN is the most common form of primary glomerular disease [15]. More than 30% of patients with IgAN will develop end-stage kidney disease within twenty years. IgAN is challenging to treat. The prognosis varies from patient to patient. Consequently, identifying the causal association between thyroid cancer and IgAN has important and practical implications for kidney protection.

MR is a research approach that evaluates the causal link between exposure and disease outcome by analyzing genetic variants related to exposure. The principle of MR is similar to that of randomized controlled trials (RCTs). In MR, the randomization variables are genetic variants. MR studies evaluate the relationships discovered by clinical observational studies and search for a novel association. Disease conditions cannot convert the sequences of the germline DNA. Therefore, MR analysis reasonably avoids reverse causation [16]. No observational evidence shows the potential causal relationship between thyroid cancer and IgAN. To test this association, we performed MR analysis [17, 18] to investigate the causal impact of thyroid cancer on IgAN.

## Methods

### Study design

**Fig. 1:**
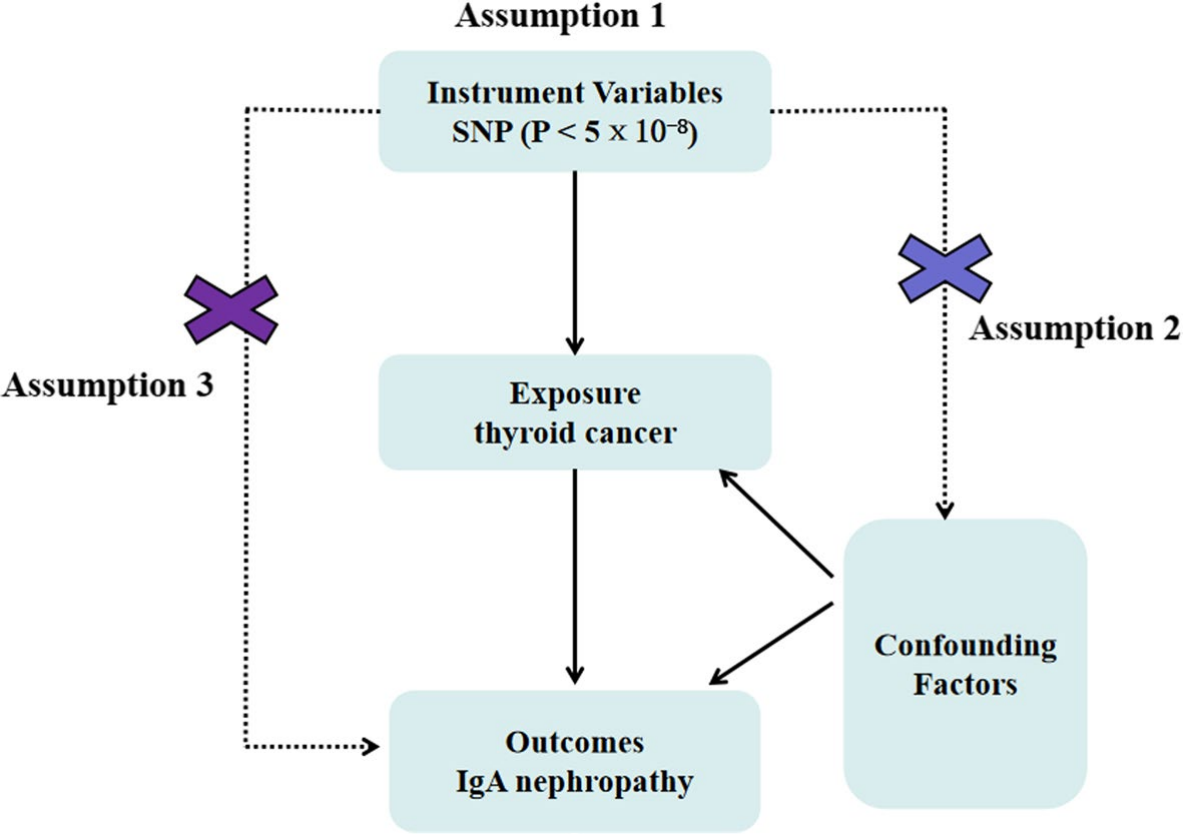
Mendelian randomization model of thyroid cancer and risk of IgA nephropathy. The design is under the assumption that the genetic variants are associated with thyroid cancer, but not with confounders, and the genetic variants influence IgA nephropathy only through thyroid cancer. SNP indicates single nucleotide polymorphism

This MR study estimated the causal influence of thyroid cancer on IgAN risk by GWAS summary statistics (Fig. 1). The authors declare that all data in this study are available. MR is a method to test the causal impact of exposure on disease development. The instrumental variables are genetic variations. This method overcomes unmeasured confounding to make some causal inferences more precise [19]. MR design is dependent on three assumptions: (1) the genetic variants are strongly correlated with thyroid cancer; (2) the genetic variants are not related to any confounders of the thyroid cancer-outcome association; and (3) the genetic variants are only related to IgAN via thyroid cancer [18].

### GWAS summary statistics for thyroid cancer and IgAN

We searched for thyroid cancer-related traits in a large-scale genome-wide association study database (GWAS) (https://gwas.mrcieu.ac.uk/datasets/ieu-a-1082/) and for available GWAS summary statistics. Before conducting MR analysis, we strictly screened single nucleotide polymorphisms (SNPs) to guarantee quality. The thyroid cancer dataset came from the Italian population. The included population was 43 to 56 years old. The IgAN dataset was a cohort of IgAN patients selected from the UK Glomerulonephritis DNA Bank. Thus, the characteristics of the two population cohorts were not similar in clinical and demographic characteristic (such as age, gender, race, education, etc.), but exists genetic comparability because they share a common European ancestry, we think they share the similar genetic profile. There was no sample overlap between the exposure and outcome datasets. First, we selected SNPs associated with the appropriate exposure at the genome-wide significance threshold (*p* < 5 × 10^−8^). Second, we aggregated SNPs in linkage disequilibrium clumping (*r*^2^ < 0.01 within windows 1000 kb for variants in the locus). Third, we calculated the F statistics of the SNPs selected above. To avoid the bias of weak instrumental variables on the final results, we excluded SNPs with F statistics less than 10. We comprehensively searched the risk factors for IgAN from previously published literature [20]. According to the previously mentioned MR assumption, we excluded SNPs associated with IgAN or the risk factors for IgAN by searching SNP information in the PhenoScanner V2 web (http://www.phenoscanner.medschl.cam.ac.uk/). The summary statistics data about the association between thyroid cancer-related SNPs and IgAN were derived from the GWAS database (https://gwas.mrcieu.ac.uk/datasets/ieu-a-1081/; ICD: “ieu-a-1081”). Ethical approval was obtained from relevant institutional review boards for the study data contributing to these GWAS meta-analyses. In the present study, we only summarized data from these studies. Therefore, it was unnecessary to require additional ethics approval.

### Statistical analysis

The IVW method can provide a consistent assessment of the causality of the exposure when each variant satisfies all three assumptions of valid instrumental variables. An estimate of IVW can be obtained by calculating the slope of the weighted linear regression. We recognized IVW as the primary approach. Two other methods (weighted median estimator and MR‒Egger) analyzed additional sensitivity because all instrumental variables correspond to the MR assumptions in the IVW method. The weighted median estimator could supply a consistent causal assessment when more than half of the instrumental variables are valid. The MR‒Egger estimation is unbiased if the genetic instrument is independent of the pleiotropic effects. We used penalized weighted median, weighted mode methods and MR‒Egger methods for additional sensitivity analyses, which make differing assumptions for the triangulation of evidence. The penalized weighted median estimator provided a consistent causal assessment when the valid instrumental variables were more than half. Furthermore, the pleiotropy and heterogeneity of SNPs were individually assessed by IVW methods with MR‒Egger intercept and Cochran’s Q statistics. We calculated the F statistics of the selected SNPs to detect the strength of the IVs at a threshold of *F* > 10, which is a typical approach in MR analysis. The R^2^ and F statistics of each SNP in the included exposure group (instrumental variable) were calculated based on previously published literature [21]. The R^2−^specific calculation formula is as follows: *R*^2^ = 2 × minor allele frequency (MAF) × (1-MAF) × beta.exposure^2^, where R^2^ is the proportion of variance explained in the instrument. The F-statistics calculation is derived from the formula F = beta.exposure^2^/standard error.exposure^2^. The effect estimates of genetically predicted thyroid cancer on IgAN are presented as odds ratios (ORs) with their 95% CIs per 1-unit-higher log-odds of thyroid cancer. The association of each SNP with thyroid cancer was further plotted against its effect on the risk for IgAN. A nonsignificant difference between the intercept and zero (*p* > 0.05) indicates the absence of pleiotropic effects. The value of Cochrane’s Q was used to evaluate the heterogeneity. If the *p* value of Cochrane’s Q was less than 0.05, the primary outcome was the IVW method with a random-effects model; otherwise, the fixed-effects model was the primary outcome. In addition, we applied the leave-one-out analysis to estimate the robustness of the results in MR analysis through any outlier SNP. If the results met the following three conditions, the causal relationship was significant: 1) the *p* value of IVW was less than 0.05; 2) the direction of the estimates among the IVW, MR‒Egger, and weighted median methods was consistent; and 3) the *p* value of the MR‒Egger intercept test was more than 0.05. We analyzed all statistics by the “two sample MR” package in R version 3.4.2 (R Foundation for Statistical Computing, Vienna, Austria). A two-tailed *p* value < 0.05 indicated statistical significance.

## Results

### Instrumental variables for thyroid cancer on IgAN

Table 1 presents the essential characteristics of the dataset included in this study.

**Table 1:** Descriptions for data sources and assessment of the instrumental variables strength

Exposures	Data sources (ID)	Race	Sample size	Cases	Controls	Access link
thyroid cancer	United Kingdom Biobank (ieu-a-1082)	Europeans	1,080	649	431	https://gwas.mrcieu.ac.uk/datasets/ieu-a-1082/
IgA nephropathy	United Kingdom Biobank (ieu-a-1081)	Europeans	5,957	977	4,980	https://gwas.mrcieu.ac.uk/datasets/ieu-a-1081/

**Fig. 2:**
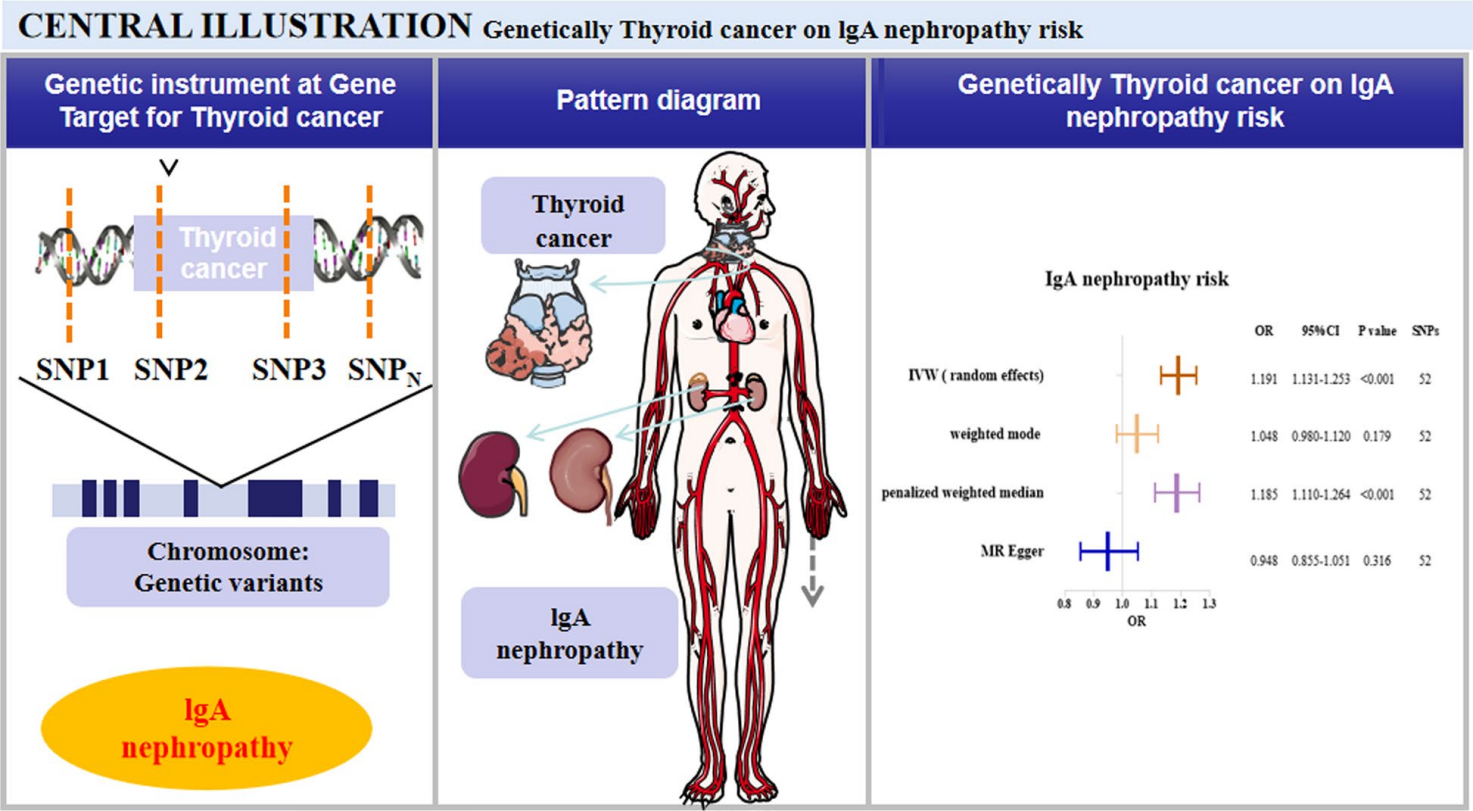
Mendelian randomization model of thyroid cancer and risk of IgA nephropathy. The overall design and abstract of the results of this study

A total of 52 available SNPs independently associated with the genetic risk for thyroid cancer were screened through previously defined SNP screening criteria (*r*^2^ < 0.01 within windows 1000 kb for variants in the locus and a *p* value < 5 × 10^−8^). We calculated the F statistics of each selected SNP to exclude weak instrumental variable bias. None of these 52 SNPs were identified as having weak instrumental variable bias, and all had F statistic values greater than 10. After analyzing the 52 SNPs in Phenoscanner, we found that no SNP was associated with IgAN or the potential risk factors for IgAN. Thus, there was a total of 52 SNPs that were instrumental variables for thyroid cancer and IgAN, as shown in Table 2. The SNP characteristics and F-statistic for each thyroid cancer and IgAN are shown in Supplementary Table 1. The strength of each SNP for thyroid cancer has an F-statistic value between 30.836 and 320.326, eliminating the bias of weak instrumental variables. Figure 2 shows the overall design and summarizes the results of this study.

**Table 2:** The characteristics of 52 SNPs and their genetic associations with thyroid cancer and IgA nephropathy

SNP	EAF	EA	OA	Thyroid cancer			IgA nephropathy		
				Beta	SE	P	Beta	SE	P
rs1008859	0.2747	T	G	-0.53717	0.09404	9.49E-09	0.0312845	0.13708	0.819474
rs10254361	0.1454	T	G	-0.71233	0.1184	1.10E-09	-0.239723	0.100965	0.0175809
rs10493096	0.1507	A	C	-0.683197	0.117	3.40E-09	-0.0604976	0.1082	0.576074
rs10495660	0.7483	G	T	0.609726	0.09652	2.03E-10	0.0327045	0.0914827	0.720722
rs10520045	0.2551	T	G	-0.588067	0.09571	6.35E-10	-0.21573	0.0991696	0.0296033
rs10762573	0.7425	C	A	0.768302	0.09634	7.78E-16	0.0680407	0.0835093	0.415204
rs10859005	0.309	T	G	-0.586627	0.09154	1.19E-10	-0.15647	0.089658	0.0809525
rs10895487	0.2081	T	G	-0.581964	0.1018	8.64E-09	-0.0233751	0.0902409	0.795612
rs11036050	0.3838	A	C	-0.515336	0.08632	2.09E-09	0.0257942	0.0866826	0.76603
rs11132917	0.6498	G	T	0.555997	0.0875	1.77E-10	-0.0115185	0.0911902	0.899484
rs11151652	0.1199	A	C	-0.835633	0.1294	4.55E-11	-0.300958	0.109432	0.00595621
rs11688848	0.1695	A	C	-0.79518	0.1122	6.33E-13	-0.0808108	0.114332	0.479686
rs11695782	0.1593	A	C	-0.640365	0.114	1.37E-08	-0.295883	0.117981	0.0121456
rs11930453	0.6681	C	A	0.55026	0.08875	4.78E-10	-0.143163	0.152277	0.347143
rs12193181	0.6422	G	T	0.575898	0.08764	4.16E-11	0.145377	0.0883949	0.100045
rs12782349	0.3045	A	C	-0.555649	0.09054	6.99E-10	-0.192955	0.0880562	0.0284315
rs1388492	0.6763	T	C	0.530178	0.08976	3.00E-09	0.0872596	0.092312	0.344522
rs1549983	0.671	G	T	0.599657	0.0891	1.34E-11	0.102053	0.0861123	0.235973
rs1555257	0.3076	T	G	-0.538026	0.09097	2.84E-09	-0.3236	0.0933796	0.000529395
rs17098351	0.7361	G	T	0.558616	0.09444	2.73E-09	0.31231	0.0916394	0.000654305
rs17600706	0.223	T	G	-0.570753	0.09982	8.72E-09	-0.0397969	0.098329	0.685675
rs1813617	0.3482	A	C	-0.510826	0.0875	4.64E-09	-0.297878	0.0878002	0.000692102
rs1873886	0.2338	A	C	-0.57412	0.09824	4.10E-09	-0.116204	0.0884691	0.189014
rs1880256	0.7554	C	A	0.632805	0.0972	5.46E-11	0.0544296	0.0897816	0.544352
rs1898422	0.1705	A	C	-0.687762	0.1108	3.50E-10	-0.010733	0.110816	0.922842
rs1951094	0.4632	T	G	-0.469524	0.0843	2.35E-08	-0.169162	0.0850833	0.0467897
rs1983033	0.2848	A	C	-0.59602	0.09325	1.29E-10	-0.116139	0.0901223	0.197508
rs2209258	0.2064	A	C	-0.642454	0.1031	3.27E-10	-0.226482	0.106358	0.0332185
rs2452477	0.1425	T	G	-0.66456	0.119	1.60E-08	-0.512627	0.128111	6.30E-05
rs2830028	0.1767	T	G	-0.72629	0.1102	2.49E-11	-0.193048	0.0973189	0.0472933
rs2885280	0.2278	A	C	-0.59602	0.1008	2.66E-09	-0.227863	0.102594	0.0263494
rs2920228	0.7538	C	A	0.618225	0.09675	1.24E-10	0.182584	0.0949094	0.0543839
rs3594	0.3292	A	C	-0.644167	0.08906	3.47E-13	-0.0977765	0.0880197	0.266634
rs4588797	0.234	T	G	-0.641314	0.09854	5.43E-11	-0.0503889	0.0959563	0.599497
rs4760566	0.8155	C	A	0.60991	0.1081	1.25E-08	0.0451727	0.0957276	0.637007
rs4792390	0.3199	A	C	-0.776964	0.09028	3.96E-18	-0.214177	0.0933952	0.0218343
rs4904753	0.2344	T	G	-0.613228	0.09848	3.58E-10	-0.301536	0.162303	0.0631888
rs4909666	0.3804	A	C	-0.550086	0.08652	1.74E-10	-0.272714	0.0887069	0.00210979
rs494791	0.7614	C	A	0.573056	0.09802	4.03E-09	0.148853	0.0905218	0.100096
rs6541725	0.3541	A	C	-0.582143	0.08777	2.70E-11	-0.282959	0.0901595	0.00169852
rs6715968	0.8038	G	T	0.728774	0.1059	3.30E-12	0.192013	0.103612	0.0638557
rs6884647	0.4009	T	G	-0.481429	0.08526	1.50E-08	-0.0906695	0.0856033	0.289517
rs6937429	0.6614	G	T	0.531369	0.08824	1.49E-09	0.0627157	0.084805	0.459586
rs7000123	0.153	A	C	-0.649896	0.1158	1.39E-08	-0.29283	0.114239	0.0103679
rs7018634	0.1281	A	C	-0.703804	0.125	1.14E-08	-0.148741	0.127767	0.244358
rs745213	0.8226	G	T	0.621013	0.1091	9.31E-09	0.426973	0.119639	0.0003586
rs7923523	0.2395	A	C	-0.542316	0.09766	2.35E-08	-0.172738	0.102637	0.0923762
rs8095811	0.05434	G	T	-2.12528	0.2471	6.04E-24	-0.211243	0.133393	0.113281
rs950275	0.2718	A	C	-0.869407	0.09534	2.65E-20	-0.3697	0.100892	0.000247999
rs965513	0.6011	G	A	-0.592221	0.08694	7.92E-12	-0.125988	0.0858024	0.14201
rs971732	0.2351	C	A	-2.13877	0.1195	1.06E-85	0.0284921	0.0789103	0.718047
rs9848511	0.1511	T	G	-0.719286	0.1172	5.08E-10	-0.15599	0.11662	0.18103

*EAF* effect allele frequency, *EA* effect allele, *OA* other allele

### The causal effect of thyroid cancer on IgAN

**Fig. 3:**
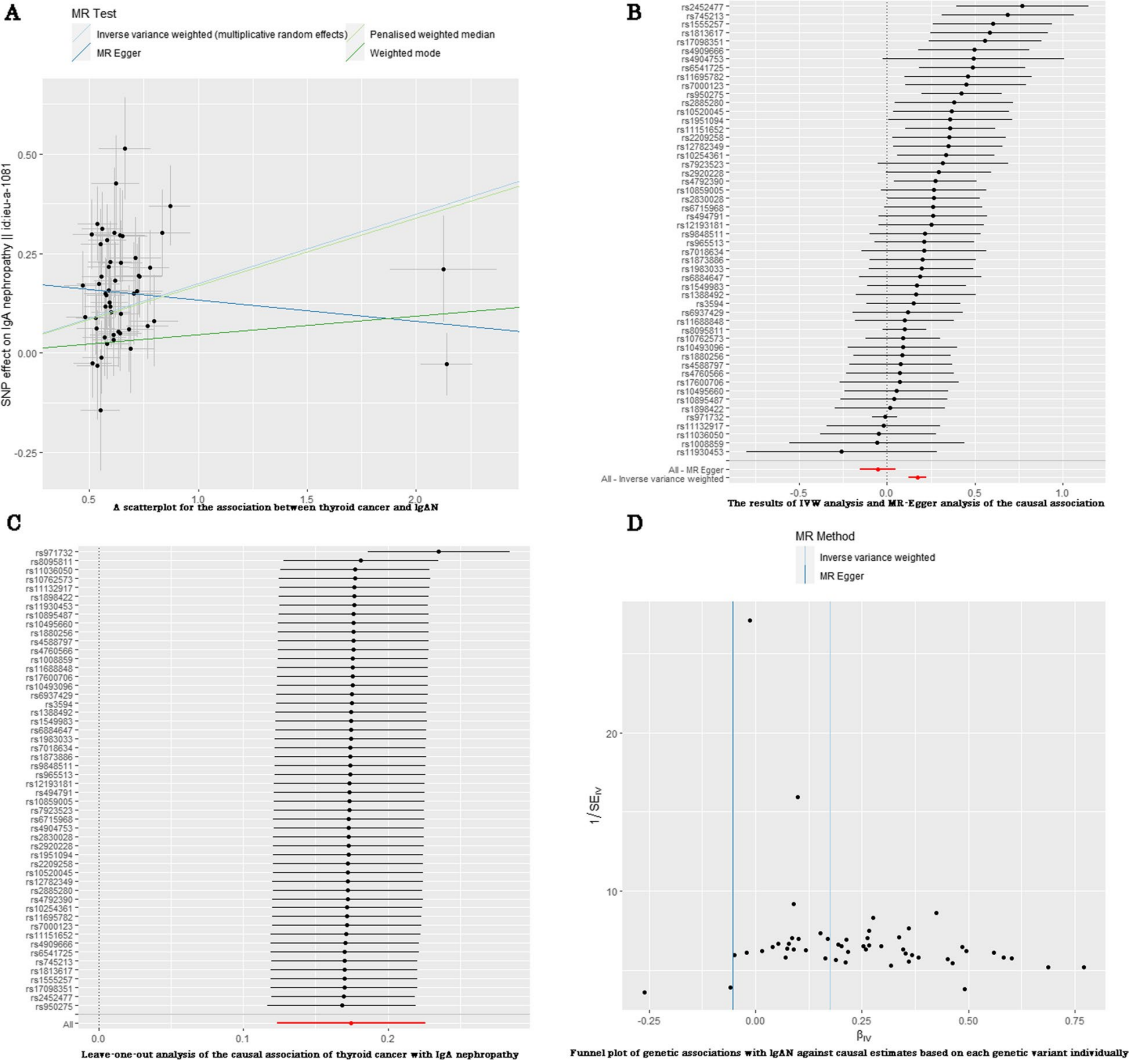
**A** Scatter plot to visualize the causal effect of thyroid cancer on IgA nephropathy. Scatter plots of genetic associations with thyroid cancer against the genetic associations with IgA nephropathy. The slopes of each line represent the causal association for each method. The blue line represents the inverse-variance weighted estimate, the red line represents the weighted median estimate. **B** Fixed-effect IVW analysis of the causal association of thyroid cancer with IgA nephropathy. The black dots and bars indicated the causal estimate and 95% CI using each SNP. The red dot and bar indicated the overall estimate and 95% CI meta-analyzed by MR-Egger and fixed-effect inverse variance weighted method. **C** MR leave-one-out sensitivity analysis for thyroid cancer on IgA nephropathy. Circles indicate MR estimates for thyroid cancer on IgA nephropathy using inverse-variance weighted fixed-effect method if each SNP was omitted in turn. **D** Funnel plot of genetic associations with IgA nephropathy against causal estimates based on each genetic variant individually, where the causal effect is expressed in logs odds ratio of IgA nephropathy for each unit increase in thyroid cancer. The overall causal estimates (β coefficients) of thyroid cancer on IgA nephropathy estimated by inverse-variance weighted (light blue line) and MR-Egger (navy blue line) methods are shown

We performed four MR methods, including the inverse-variance weighted model, weighted mode, penalized weighted median, and MR‒Egger method, to analyze these data (Fig. 3A). Thyroid cancer significantly increased the risk of IgAN in the random-effect IVW model (OR, 1.191; 95% CI, 1.131–1.253, *P* < 0.001), as shown in Fig. 2 and Table 3. This causal link was also observed in the weighted mode method (OR, 1.048; 95% CI, 0.980–1.120, *P* = 0.179) and penalized weighted median (OR, 1.185; 95% CI, 1.110–1.264, *P* < 0.001) (Fig. 2, Table 3). However, the MR‒Egger method showed that thyroid cancer nonsignificantly decreased IgAN risk (OR, 0.948; 95% CI, 0.855–1.051, *P* = 0.316) (Fig. 3B). Therefore, based on our previous interpretation of the results, our findings did not suggest a robust and reliable association between thyroid cancer and IgAN.

**Table 3:** The Association of thyroid cancer with IgA nephropathy risk using various methods

Method	Beta	SE	OR	95% CI	Z score	*P* value
IVW (random effects)	0.175	0.026	1.191	1.131–1.253	6.628	< 0.001
MR Egger	-0.053	0.053	0.948	0.855–1.051	-1.014	0.316
Weighted mode	0.046	0.034	1.048	0.980–1.120	1.370	0.179
Penalised weighted median	0.169	0.033	1.185	1.110–1.264	5.088	< 0.001

*OR* odds ratio, *CI* confidence interval, *IVW* inverse variance-weighted, *MR* Mendelian Randomization

### Evaluation of assumptions and sensitivity analyses

In addition, IVW analysis (Q = 105.414, *P* = 0.020) and MR‒Egger analysis (Q = 72.567, *P* < 0.001) were performed to determine heterogeneity. MR‒Egger regression revealed the directional pleiotropic effect across the genetic variants (intercept, 0.186; *P* < 0.001). After deleting each SNP, the result of the merger of the remaining SNPs is basically in a straight line. Thus, no single SNP significantly impacted the MR estimation results based on leave-one-out analysis (as shown in Fig. 3C), with all significant estimates ranging from 0.16 to 0.24. Furthermore, asymmetry in the funnel plot can bias MR methods by indicating directional horizontal pleiotropy. Thus, we examined the funnel plot for asymmetry but found no such evidence in our study (as shown in Fig. 3D).

## Discussion

This MR is the first study to explore the causality of thyroid cancer on IgAN risk.

Although IVW was the primary method that demonstrated the impact of thyroid cancer on IgAN, a contrary direction in MR‒Egger did not support this association. Therefore, the evidence in this study does not sufficiently demonstrate that thyroid cancer increases the genetic risk for IgAN.

Onconephrology is a new field that has appeared during the last few years [22]. A large-scale renal disease emerges in patients with cancer. Kidney injury may result from cancer treatment, chemotherapeutic drugs, and malignancy. Currently, nephrologists encounter an increasing number of cancer patients with clinical features manifesting as nephropathy syndrome. They confront a barrier in the treatment of these patients. Identifying whether nephropathy syndrome is derived from malignancy or chemotherapeutic drugs is complex. This uncertainty creates hesitation in discontinuing chemotherapeutic drugs or starting kidney treatment [23–26]. Nephropathy syndrome treatment always requires glucocorticoid or immunosuppressant administration [27, 28]. However, these pharmaceuticals induce many adverse drug reactions, including infection, osteoporosis, diabetes, and gastrointestinal reaction [29]. If a cancer patient suffers from kidney disease caused by malignancy, withdrawal of chemotherapeutics or the initiation of glucocorticoids/immunosuppressants is ineffective and worsens the patient’s condition. Antineoplastic therapy is much more crucial than kidney protection. Stopping antitumor treatment may threaten survival. Therefore, identifying kidney injury induced by cancer is essential [30].

Recently, some cases of nephropathy syndrome induced by solid tumors have been reported. Most solid tumors associated with membranous nephropathy are lung and gastric cancers, followed by prostate cancer, thymoma, and so on [31]. Minimal change disease is frequently observed in lung, colorectal, and thymoma and rarely in pancreatic, bladder, breast, and ovarian cancers [32]. The relationship with IgAN occurs in the respiratory tract, buccal mucosa, and nasopharynx [33]. These findings indicate various pathological types of nephropathy syndrome induced by solid tumors. However, whether thyroid cancer increases the risk of nephropathy syndrome has not been confirmed. Thyroid function has a vital effect on kidney development. Thyroid dysfunction directly worsens kidney function and leads to the development of kidney disease. A 52-year-old woman was diagnosed with nephropathy syndrome resulting from medullary thyroid carcinoma [14]. Clinicians discovered diffuse glomerular deposition of amyloid by kidney biopsy. Medullary thyroid carcinoma releases the calcitonin hormone, forming amyloid deposits in the kidney. This case indicates that thyroid cancer probably causes injury to the glomerulus. This MR did not ultimately confirm a causal link between thyroid cancer and IgAN. Despite insufficient evidence supporting the notion that thyroid cancer patients tend to develop a risk of IgAN, we cannot ignore the probability that thyroid cancer has a potential influence on the kidney. We discovered some connections between thyroid cancer and IgAN in previous reports. We discussed that these associations act through the underlying mechanism of circulating immune complexes and complement activation.

First, thyroid antigens were in the glomerular deposits. Thyroid antigen–antibody probably causes the deposition of circulating immune complexes in the kidney. Immune complexes thicken the glomerular basement membrane, alter podocyte function, and activate the classic pathway of the complement system. Complement system activation accelerates the inflammatory process through the chemotactic factors C3a and C5a. According to previous evidence, glomerular inflammation is one of the mechanisms involved in IgAN [34]. We speculate that the deposition of thyroid antigen–antibody circulating immune complexes increases the risk of IgAN. Furthermore, this assumption is consistent with previous evidence [35]. According to Santoro et al., autoantibodies directed against the epitopes of thyroglobulin, thyroperoxidase, and glomerular antigens likely cause immune-mediated glomerular disease. Additionally, epitope spreading has not yet been studied in patients but has been shown to occur in experimental immunization with an immunogenic thyroglobulin peptide. Second, previous studies established that thyroid cancer cells could produce IgG. IgG is positively associated with the growth and metastasis of thyroid cancer cells. In thyroid cancer tissues, the colocalization of IgG with C1q, C3c, and C4c was observed [36]. IgG was also detected in glomerular immune deposits of all IgAN patients [37]. IgAN is an autoimmune disease characterized by the glomerular deposition of immune complexes [38–40]. In patients with IgAN, the circulating immune complexes consist of IgG, IgA, IgM, and complement C3 [41, 42]. C3 is biologically capable of activating the complement pathway [43] and is present in kidney biopsy specimens of patients with IgAN. In thyroid cancer tissues, researchers discovered C3c, a fragment of complement component C3. Further validation was required to validate the hypothesis that IgG and C3 released by thyroid cancer cells expose patients to IgAN risk. Finally, we found that IgG and TgAb IgG in thyroid cancer activate the alternative pathway (AP) and lectin pathway (LP), respectively. Some evidence suggests AP and LP as pathogenic mechanisms in IgAN through the promotion of complement activation. AP and LP activity produce a pathogenic link between glomerular IgA deposition and glomerular inflammation and injury [34]. Patients with thyroid cancer likely have a risk of AP and LP action, which stimulates the complement to induce kidney injury in IgAN. However, this assumption will have to be demonstrated by future studies. Animal experiments are still important methods for investigating and validating these assumptions in the future.

## Conclusion

In conclusion, according to the MR principle, the evidence of this study did not confirm a stable significant causal association between thyroid cancer and IgAN. However, based on previous studies, we cannot neglect the potential connection between thyroid cancer and IgAN from the perspective of circulating complex immune depositions and complement activation. This assumption and underlying impact deserve more investigation and exploration.

### Limitations

This study still has several limitations. First, enrolled participants were of European ancestry. Thus, the results are not applicable for other ethnic groups. Second, MR analyses established causal hypotheses by randomly distributing genetic variants. It was not easy to differentiate mediation and pleiotropy by the MR technique. In our genome, huge variants probably influenced one or more phenotypes. Third, additional mediator methods and observational approaches cannot validate the metabolic pathways underlying the link between thyroid cancer and IgAN. Future research will examine the underlying process since the UK Biobank data have limitations. It will provide valuable recommendations for clinical practice.

### Supplementary Information

**Additional file 1.**

**Additional file 2.**

## Data Availability

All data generated or analyzed during this study are included in this published article.

## Abbreviations

IgAN: IgA nephropathy
MR: Mendelian randomization
IVW: Inverse variance-weighted
(GWAS): Genome-wide association studies database
OR: Odds ratio
Cl: Confidence interval
AP: Alternative pathway
LP: Lectin pathway
